# How to orchestrate a soccer team: Generalized synchronization promoted by rhythmic acoustic stimuli

**DOI:** 10.3389/fnhum.2022.909939

**Published:** 2022-07-29

**Authors:** Manfred A. Müller, Antonieta Martínez-Guerrero, Maria Corsi-Cabrera, Alfred O. Effenberg, Armin Friedrich, Ignacio Garcia-Madrid, Matthias Hornschuh, Gerd Schmitz, Markus F. Müller

**Affiliations:** ^1^Centro Internacional de Ciencias, A.C., Cuernavaca, Mexico; ^2^Instituto de Ciencias Básicas y Aplicadas, Universidad Autónoma del Estado de Morelos, Cuernavaca, Mexico; ^3^Sleep Laboratory, Faculty of Psychology, Universidad Nacional Autónoma de México, Mexico City, Mexico; ^4^Institute of Neurobiology, Universidad Nacional Autónoma de México, Queretaro, Mexico; ^5^Leibniz Universität Hannover, Institut für Sportwissenschaft, Hannover, Germany; ^6^Posgrado en Ciencias Sociales, Facultad de Estudios Superiores de Cuautla, Universidad Autónoma del Estado de Morelos, Cuautla, Mexico; ^7^Institut für Musik und Musikwissenschaft, Stiftung Universität Hildesheim, Kulturcampus Domäne Marienburg, Hildesheim, Germany; ^8^Centro de Investigación en Ciencias, Universidad Autónoma del Estado de Morelos, Cuernavaca, Mexico; ^9^Centro de Ciencias de la Complejidad, Universidad Nacional Autónoma de México, Mexico City, Mexico

**Keywords:** interpersonal coordination, entrainment, dynamic attention theory, tempo preference, rhythmic acoustic stimuli and cognition, generalized synchronization

## Abstract

Interpersonal coordination requires precise actions concerted in space and time in a self-organized manner. We found, using soccer teams as a testing ground, that a common timeframe provided by adequate acoustic stimuli improves the interplay between teammates. We provide quantitative evidence that the connectivity between teammates and the scoring rate of male soccer teams improve significantly when playing under the influence of an appropriate acoustic environment. Unexpectedly, female teams do not show any improvement under the same experimental conditions. We show by follow-up experiments that the acoustic rhythm modulates the attention level of the participants with a pronounced tempo preference and a marked gender difference in the preferred tempo. These results lead to a consistent explanation in terms of the dynamical system theory, nonlinear resonances, and dynamic attention theory, which may illuminate generic mechanisms of the brain dynamics and may have an impact on the design of novel training strategies in team sports.

## Introduction

Team sport implies teamwork. This requires precise interpersonal coordination in a common timeframe, oftentimes with scarce or even without any verbal communication. In the field of dynamical systems, the behavior where two dynamical units perform differently but one in function of the other is termed as “generalized synchronization” (Rulkov et al., 1995) and is quantified by statistical interdependencies between corresponding phase spaces (Arnhold et al., 1999).

Usually, synchronization is understood as “doing the same thing at the same time” (complete 1:1 synchronization) or at least doing the same thing a time lag apart from (lag synchronization). However, modes like (2:1, 3:1, 7:8, in general m:n) are possible and realized (refer to, e.g., Hasson and Frith, 2016). In the cases, two systems with the same instantaneous frequency orbit the attractor, or one unit with an integer multiple of the frequency of the other.

However, more complex scenarios with more complex attractor topologies are also conceivable, in which the movement in the phase space of one system is coupled to that of another, so there is a functional relationship between both movements in phase spaces: $\vec{X}\,\left(t\right)=\vec{F}\,\left(\vec{Y}\,\left(t\right)\right)$. In this case, the temporal evolution of the two systems, i.e., their dynamics, may be completely different, but they are not independent of each other. That is, if one system is in a particular position in its phase space at a particular time, it implies that the other system is in a specific region in its phase space at the same time, i.e., the positions of both systems in their respective phase spaces are strictly correlated and, therefore, their respective dynamics. If we denote the phase spaces of each of the systems by *DX* and *DY*, the common phase space of the coupled systems is given by *DX*×*DY* (note that this space is much larger than just the sum of *DX* and *DY*). Nevertheless, there is dynamic compression (Riley et al., 2011, Araújo and Davids, 2016). Although the coupled phase space is significantly larger than both uncoupled spaces, the number of degrees of freedom of each subsystem is reduced by the coupling. The larger flexibility of each subsystem provided by the enlarged space DX × DY is reduced by the functional coupling and thus, ensures the stability of the coupled system (Gorman et al., 2017). A second ingredient for synergy is reciprocal compensation, which means that one dynamical subunit of the coupled system react to changes of the others (Riley et al., 2011; Araújo and Davids, 2016). Given that in the extended phase space the dynamics of each subunit is strongly correlated to the others, this requisite is automatically fulfilled for coupled nonidentical complex systems. Only the amount of correlations between subunits depends on the amount of coupling strength. This scenario is called “generalized synchronization” (Rulkov et al., 1995).

Transferring this mathematical picture to real-life situations of social systems means that the behavior of several interacting subjects, i.e., pursuing a common goal, can be strikingly different, but each one acts in function of the others. In this sense, the terms “generalized synchronization,” “joint action,” and “Synergy” (Riley et al., 2011; Araújo and Davids, 2016) are synonyms referring to the same phenomenon but are used in different scientific communities.

Prominent real-world examples of such generalized synchronization are orchestras or soccer teams. However, musicians benefit continuously from the score, the acoustic feedback of the whole ensemble and if present from the external driving of the conductor, while the coupling between teammates is much weaker and reduced to actions perceived in a limited visual sector and a restricted acoustic radius. Here, team play is promoted through clearly defined positions and functions of individual players and tactical advice from the coach. Moreover, shared intentionality might play an important role in uncertain or novel situations (Phillips-Silver et al., 2010; Araújo and Davids, 2016). However, particularly for modern association football, team play emerges largely in a self-organized manner and depends sensitively on actual game situations (Araújo and Davids, 2016). It is for this reason why we decided to take modern association football as a testing ground for a novel joint action experiment.

Precise spatiotemporal coordination is also required from the sensory-motor system. It is widely accepted that rhythmic acoustic stimuli promote motor control even in the case of patients with severe motor disorders (O’Callaghan and Turnbull, 1987; Aldridge, 1993; Thaut et al., 1993; Altenmüller and Schlaug, 2013; Ghai et al., 2018). Here, music improves particularly the precision of movement but not necessarily speed (Bernatzky et al., 2004). Specifically, when real recordings of a moving subject are provided instead of synthetic sounds, auditory stimulation improves performance in healthy subjects and patients with Parkinson’s disease (Young et al., 2014; Bailey et al., 2018; Murgia et al., 2018). Furthermore, the tempo at which the rhythm is displayed can have a critical impact on athletic performance (Pizzera et al., 2017).

The positive effect of acoustic rhythms is attributed to the orderly time structure of musical rhythms, which may act like a kind of trigger for brain structures like the cerebellum that are supposed to be responsible for timing of motor actions (Molinari et al., 2003). Although other qualities of music like groove may influence this scenario (Stupacher et al., 2013), rhythmic time structure seems to play a central role in this process.

Auditory rhythms rapidly entrain extended neural networks involved in motor acts even unconsciously (Thaut, 2003) such that the time order of motor responses is guided by the rhythmic time structure of the music and coordinates the communication of different brain areas (Thaut, 2003). In addition, current studies show that acoustic motion information can directly influence executive motor functions in addition to perceptual functions (Schmitz et al., 2013), and that it can also have effects on motor functions that go beyond rhythmic adjustments (Effenberg et al., 2016; Effenberg and Schmitz, 2018). A review of physiological, psychological, and psychophysical benefits of music in sports can be found in Terry et al. (2020).

Communication, on the other hand, is inherently rhythmical (Bispham, 2006). An interpersonal interaction is not an erratic process, where information fluxes are interchanged in a stochastic manner but occurs in an oscillating fashion, which may imply the presence of a dynamic process in the development of social interactions. The observation that a communicative interaction between 3- and 5-month-old infants and their mothers occurs in cycles may indicate that such a behavior is innate and could constitute an important precursor for language acquisition (Lester et al., 1985). Thus, the oscillatory features of social interaction of 3-week-old infants have been attributed to attention/non-attention phases (Wolff, 1967). It has even been suspected that the irregularities of interaction rhythms may have a diagnostic value and may serve as a measure of neurological functioning and early detection of communicative deficiencies (Lester et al., 1985). Finally, also, a nonverbal communication between individuals, probably a driving force of self-organized team management, displays a rhythmic time structure of interactions between the protagonists (Froese et al., 2014). The current study indicates different effects of knowledge- vs. performance-based kinds of auditory real-time feedback on interpersonal coordination in a joint task (Hwang et al., 2018).

Acknowledging that (a) musical rhythms significantly improve intrapersonal coordination and (b) social interactions (verbal and nonverbal) are inherently of rhythmic nature, we ask in this contribution if, furthermore, adequate rhythmic acoustic stimuli may also promote interpersonal coordination such that a group of individuals act like a kind of a super-organism (Duarte et al., 2012; Müller et al., 2021). That this is true in terms of perfect synchronization of movement (Roederer, 1984; McNeill, 1995), or at least for similar rhythmic motor actions (Keller, 2012), is well known. However, here, we refer to more complex, self-organized systems in terms of generalized synchronization, where people may act quite differently but everyone performs in function of the others. Whether a common rhythmic acoustic timeframe also promotes interpersonal coordination in this generalized sense is unknown so far but is considered in this study. We hypothesize that an adequate rhythmic acoustic stimulus modulates the attentional level, which, in consequence, foster interpersonal coordination. Association football is chosen here solely as a convenient testing ground to probe interpersonal coordination in a self-organized fashion without major constraints by external rules. In this sense, this is not an article about soccer, although we hope it may have implications for future training strategies. *Via* a sequence of experiments, we collect sufficient materials to propose a model for brain dynamics, which is congruent with the dynamic attention theory (Riess Jones et al., 1981; Large and Riess Jones, 1999) and nonlinear resonances; thus, we hope to contribute to the field of theoretical neuroscience.

## The soccer experiment

The experiments have been approved by the ethics committee of Centro de Investigación Transdisciplinaren Psicología of the Universidad Autónoma del Estado de Morelos, Mexico. All participants in the three experiments realized in this study have been clinically healthy at the time of experimentation, and none of them suffered from any overt perceptual or motor impairments.

### Description of the soccer experiment

In total, 52 male and 42 female players with a mean age of 18.8 ± 2.2 and 21.6 ± 4.6 years, respectively, participated. All the participants have practiced soccer regularly for at least 10 years at least two times a week and play in district to regional German soccer leagues. They all gave their written informed consent for the study. The experimental protocol was in accordance with the ethical standards of the American Psychological Association. The players were given general information on the experiment. For instance, they knew that we aim to study the influence of “music” on soccer performance, but they did not know about the particular purpose of this project. Furthermore, the players were not aware that teammates or players of the other team were under the influence of the same or a different acoustic environment.

**FIGURE 1:**
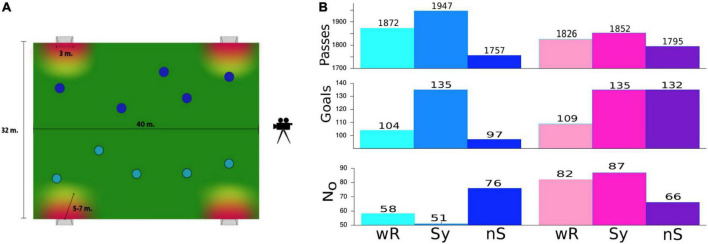
**(A)**. Sketch of the soccer field. Scoring was only allowed within a critical radius around the goal. This is a typical exercise routine, which is commonly implemented in training sessions to improve interpersonal coordination expressed by fast and precise pass sequences or, in the terminology of this study, to improve the connectivity of a team. **(B)**. Total number of passes and goals for male (blue colors, left) and female teams (pink colors, right) were obtained under the three experimental conditions. N0 denotes the number of ball possessions of a team without any pass. wR = without rhythmic acoustic stimuli, Sy = rhythmic acoustic stimuli displayed collectively in a synchronous fashion at 140 bpm, nS = rhythmic acoustic stimuli displayed collectively but each player with another tempo.

Randomly constituted teams of 5 players are advised to play on a reduced pitch (32 m × 40 m) with two goals (with a width of 3 m) on each side (Figure 1A) for a period of 30 min, which is divided in thirds of 10. Occasionally, some of the players participated several times in the experiment. The coach’s instructions as well as the whole concept of this training exercise were to practice fast and efficient passing sequences. Therefore, scoring by long distance shots was not allowed. All the players were familiar with this training exercise because it is frequently used to improve precision and timing of fast pass-sequences.

One third was played without rhythmic stimulation (wR). During two-thirds of a match, rhythmic auditory stimulation (RAS) is provided collectively (cRAS) to all the players, to one team in a synchronous (Sy) condition, to the other one in a non-synchronous (nS) condition, and vice versa. “Synchronous” means that the sound is displayed phase-synchronously at 140 bpm (Simpson and Karageorghis, 2006; Karageorghis and Priest, 2012a,b) to all the players of one team. In the non-synchronous mode, the same rhythm is presented but for each player with its own tempo (119, 133, 147, 154, and 161 bpm). All games were video-recorded for posterior quantitative analysis. In total, 16 matches with male and 14 matches with female teams were performed.

The chronological order of the three conditions was chosen in an almost balanced but randomized manner. Thirds without RAS has been applied 6 times at the beginning, 6 times in the middle, and 4 times at the end of a game for male teams; corresponding numbers for female teams are: 5, 5, and 4.

Because of the possible influence of individual music preferences on physiological performance parameters (Karageorghis and Priest, 2012a,b), we intended not to adopt any particular style. Instead, we derived the rhythm of the acoustic stimulus from the movement of a professional soccer player. Hence, we intend to stimulate soccer players with a football-affined rhythm, avoiding stylistic preferences to a maximal extent.

We used the time pattern of dribbling of South African offensive midfielder Teko Modise, a Premier Soccer League player from years 2008 and 2009. The temporal train of footsteps and ball contacts has been translated to a sequence of musical beats using the program “Logic Pro 9.” This rhythm has been instrumented and looped with changing selection of instruments such that the resulting musical piece covers the period of one-thirds of a 30-min training session. Because of two reasons, the 140-bpm rhythm was chosen as the stimulus of the synchronous mode. First, it was reported that the performance of male 400-m racers improves significantly while hearing musical rhythms of this tempo (Simpson and Karageorghis, 2006). Setting the dribbling of several male soccer players to music further motivated the choice of 140 bpm for the synchronous condition, because we found that movement patterns are narrowly distributed around this tempo. Thus, the results of the 400-m racers and the soccer players seem to be congruent.

FM radio technology was used to ensure that both teams received the musical sequence in exact temporal synchrony (temporal difference <10 ms). The music was played back using the program Logic Pro 9. An interface (Echo AudioFire 12) was used to assign 1 of the 11 parallel soundtracks (for 10 players and one camera) to each transmitter (Sennheiser SRF 300 IEM G3-E-X). Four transmitting signals were fed to a directional antenna (Sennheiser A2003) through a combiner (Sennheiser Antenna Combiner AC 3). The players received the signals *via* a mobile receiver (Sennheiser EK 300 IEM G3 E-X) and headphones. The headphones (Adidas OMX 680) were specially designed to be used while playing sports and partially allow for transmission of ambient noise. To detect performance level, all the games were recorded with a video camera (Panasonic HDC SD100 EGK), which was located outside the playing field covering the whole pitch. Ball contacts, successful passes, and the number of goals were quantified during offline analysis.

### Statistical analysis of the soccer experiment

The connectivity of a soccer team depicts the ability of the players to act together in the sense of generalized synchronization (Rulkov et al., 1995). It was introduced by the observation that nonidentical but weakly coupled chaotic oscillators may establish a nontrivial functional relationship between their dynamics, although they may not synchronize in a classical manner (doing the same thing at the same time).

Mathematically, such interrelationship is captured in a statistical sense such that instantaneous positions of the systems on the corresponding nonidentical attractors are correlated (Arnhold et al., 1999). These concepts have been generalized to many biological or social systems where a proper definition of a phase space and, hence, an adequate estimate of synchronization measures are impossible. In this spirit, movement patterns of individuals, as well as interpersonal coordination, have been frequently described in terms of dynamical systems (Schmidt and Richardson, 2008). Equivalent to nonidentical coupled chaotic oscillators, different players of a soccer team act differently, but there exists a functional relationship between players of the same team.

In the present experimental setup, effective team play is expressed by precise and fast pass sequences with a minimal number of ball contacts of each player. When the coupling between players is high, the ballcarrier knows about the position of his teammates, and his teammates try to disrupt the attacker’s defense or position themselves in an open space so they can receive the pass. Thus, a fast pass sequence requires synergy in the team where all or almost all players participate and act like a super organism. Ideally, in this training exercise where fast passing sequences are required, the reception of the ball simultaneously represents the pass to a teammate (one touch play).

In this spirit, the connectivity of a team is quantified separately for each condition by the number of passes *n*p** divided by the number of ball contact *n*c**s measured during a 10-min third *C*^*Condition*^ = (*np*/*nc*)^*Condition*^, where “condition” refers to thirds without cRAS, the synchronous or the non-synchronous acoustical environment. Note, the number of passes between two teammates has already been used in Duch et al. (2010) in a graph theoretical context. Here, we used the quotient between the number of passes and ball context to consider the efficacy of team play.

**FIGURE 2:**
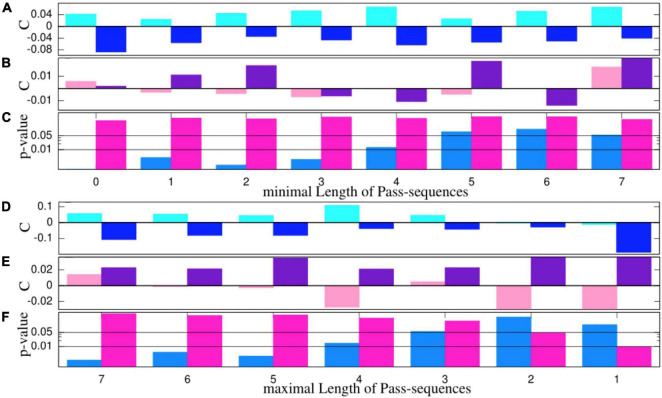
**(A,B,D,E)** Medians of relative connectivity values normalized to those estimated from the thirds without cRAS. Blue colors refer to male and pink color to female teams. Light pink/blue refers to the Sy-condition and dark pink/blue to the nS-condition. Panels **(C,F)** display the *p*-value on a logarithmic scale estimated by Mann-Whitney-Wilcoxon rank test, which provides the probability that the samples obtained for the Sy- and nS-modes stem from the same distribution. Horizontal black lines in panels **(C,F)** indicate the 1 and 5% significance level, respectively. The abscissa in panels **(A–C)** and **(D–F)** mark the minimal (maximal) length of pass sequences considered.

Here, we are interested in which manner and on what amount the performance of a given team changes under the three conditions. To get rid of the absolute strength of a team in terms of connectivity and focus solely on relative changes, we contemplate relative connectivity values, which are normalized to those estimated from thirds without cRAS: $C=\frac{C^{R\,A\,S}-C^{w\,R}}{C^{w\,R}}$. In this way, the absolute strengths of the randomly constituted teams are eliminated from the statistics, and we focus exclusively on relative changes in the quality of their team play compared to the wR-condition, e.g., estimates of 0.1 connote a 10% increase in the connectivity value of a team in a Sy or an nS condition compared to the wR-mode. Therefore, although the results presented in Figure 2 refer solely to the Sy- and nS-modes, the sign of the empirical estimates indicate additionally the comparison with the wR-condition. Of note, the difference between the relative connectivity values obtained for the Sy and nS settings can be understood as a kind of effect size. Significance values are estimated by nonparametric Mann-Whitney-Wilcoxon rank test.

**FIGURE 3:**
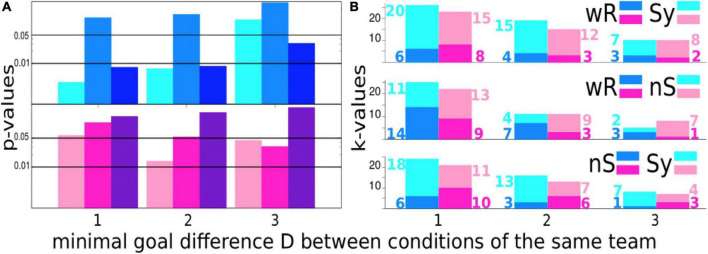
**(A)** Probabilities that the scoring differences D of a team playing under different conditions occur by chance according to the binomial distribution for the male (upper panel) and female teams (lower panel) on a logarithmic scale. We compare the setting without rhythmic stimulation (wR) with the synchronous displayed condition (Sy) (light pink/blue), wR- vs. the non-synchronous acoustic stimulation (nS-) condition (pink/blue) and the Sy-condition vs. nS-condition (dark pink/blue). Horizontal black lines indicate the 1 and 5% significance level. **(B)** Number of times where the scoring difference was in favor of one or the other condition. Each panel shows the comparison of two conditions for different values of the scoring difference D. Again, blue colors denote results for the male and pink colors for the female teams. The color code for the settings wR, Sy, and nS is indicated in the right border at each panel. wR = without rhythmic acoustic stimuli, Sy = rhythmic acoustic stimuli displayed collectively in a synchronous fashion at 140 bpm, nS = rhythmic acoustic stimuli displayed collectively but each player with another tempo.

To create Figure 3, we proceed with the same philosophy as before, *viz*., we compare the performance of the same team under different conditions. To this end, we counted the number of times when the scoring difference of a team under different conditions is at least D (Figure 3B) and, further, how many times the scoring difference has been observed in favor of one or the other condition, e.g., comparing wR- and Sy-condition *kwR* = 6 times with a scoring difference of at least 1 was quoted in favor of wR and *kSy* = 20 times in favour of Sy (Figure 3B, upper panel). If one assumes the null hypothesis that scoring differences between conditions appear with equal probability *pwR* = *pSy* = 0.5, one might estimate the probability that our results occur by chance using the binomial distribution

$$P=\left(\begin{array}{c} k\,w\,R+k\,S\,y\\ k\,S\,y \end{array}\right)\,p\,w\,R^{k\,w\,R}\,p\,S\,y^{k\,S\,y}.$$

For the abovementioned number of comparisons of the wR and Sy-conditions, this probability is amazingly small with *p* = 0.003431.

To corroborate the significance of our experimental results obtained for connectivity values and scorings, we also applied a parametric test framework probing whether the average values of the samples are statistically equivalent. To this end, we conducted first a two-sample student *t*-test on the relative connectivity values shown in Figure 2 (refer to Supplementary material). Thus, we reproduce almost quantitatively the results obtained by the nonparametric Mann-Whitney-Wilcoxon rank test (Figures 2C,F).

For the scoring statistics, we conducted a paired *t*-test where pairs of scoring rates are formed for the same team playing under different conditions (see Supplementary Figure 2). Again, we reproduced almost quantitatively the results obtained for the nonparametric statistics, namely, in the present case, the estimates for the binomial distribution (Figure 3A).

## Results of the soccer experiment

In the first step, we considered two quantities commonly used for evaluating the dominance of a soccer team, namely, the number of passes played and goals scored. As a third quantity we introduce N0, the number of ball possessions of a team without making any pass and serves as an indicator of team segregation.

For the male teams, we obtained for the Sy-condition highest number of passes and goals and simultaneously reduced number of ball possessions without a pass, while for the nS-mode all the three statistics indicate poorest performance. Notable is the high increase in scores of about 30% in the Sy-condition as well as the 30% increase in N0 for the nS-condition for the male teams. For the female teams, the number of passes is highest for the synchronous and lowest for the nonsynchronous conditions, although the difference between the conditions is less pronounced. Scoring was notably higher in both conditions with cRAS (again about 30% higher than without RAS), while team segregation seems to be highest in the Sy-mode and lowest in the nS-condition. The reduction of N0 in the nS mode in comparison with the Sy-mode is again about 30%. Although this is not the main focus of this study, we also compared the performance between genders. For this purpose, we normalized the numbers in Figure 1 according to the teams’ playing time (the total number of matches was different for the male and female teams). A corresponding table is included in Supplementary material. Here, it can be seen that the indicators of the female teams are consistently higher than those of the male teams for all the three indices.

In summary, we obtained consistent results for the male teams, indicating a trend clearly in favor of the Sy- and lowest performance of the nS-condition, while the results for the female teams are puzzling and inconsistent. However, the results presented in Figure 1 lack any objective significance test and are, thus, not conclusive.

As a step toward more substantiated statistics, we evaluate the interpersonal coordination or the connectivity of a team. Here, we are interested in which manner and on what amount the performance of a given team changes under the three conditions. For this reason, here, we refer to relative connectivity values with respect to the condition without cRAS. In this way, numerical estimates do not depend on the actual strength of the randomly constituted teams, but positive/negative relative values provide the percentage of the improvement/worsening of a team playing under the condition of cRAS with respect to the condition without music. The results are summarized in Figure 2.

The median relative connectivity values estimated for the male teams in the Sy-condition are systematically higher than those estimated without cRAS and are, in turn, above the medians estimated for the nS-condition in all the cases shown in Figure 2A. If all pass sequences are considered (the two very left boxes in panel A), we will obtain a *p*-value of 8.7*x*10^−4^ for distinction of the Sy and nS-modes for the male teams. In that case, we observe an increase of 4% for the Sy-mode and a decrease of about 8% for the nS-mode. Only if exclusively short pass sequences are examined (*n* < 3), the connectivity values derived for the synchronous mode decrease to values slightly below the estimates obtained for the wR-condition (Figure 2D). Including solely pass sequences with at most three passes, the *p*-value is slightly above the 5% level, and it grows further for shorter sequences (Figure 2F). The reason for this drop in significance level could be that a player, contrary to the instructions and the aim of the training exercise, takes the opportunity to dribble to one of the goals, possibly with the support of his teammates. Otherwise, given the specific training situation, it is more conceivable that this could also indicate a significant loss of connection between the players.

Also, if one dissects merely large pass sequences with *n >* 5 (Figures 2A,C), we notice a loss of significance. Again, one may question if in general sequences with more than 5 passes played on a reduced field within teams of only 5 players reflect synergy in a team. It is more conceivable that straight collective attacks are played quickly in approximately 3 to 5 passes. Otherwise, the opposing defense might intervene its forward action such that the team gets forced to move backward and the global stability of the synergic dynamical state gets disturbed (Riley et al., 2011; Araújo and Davids, 2016), which leads to decrease in connectivity index during the subsequent phase of reorientation before it may increase for even longer pass sequences. Consistently, for *n* = 7, the *p*-value already decreases again. Of note, the negative trends of the indicators when playing with RAS are expectable, acknowledging that the headphones conspicuously diminish the acoustical coupling between players. Naturally, such disturbances are absent in the mode without RAS. Thus, the clear positive results for the Sy-mode for the male teams are more surprising.

The results summarized in Figure 2 pinpoint that the male teams are significantly more connected and less segregated in the synchronous mode, and that a non-synchronous cRAS promotes team segregation and impedes interpersonal space-time coordination. For the female teams, on the other hand, the results are puzzling and qualitatively different. The connectivity values obtained for, the Sy-mode and the nS-mode take occasionally positive and negative values in an irregular fashion without showing any systematic trend, and the significance values are far outside the 5% limit, which depicts a striking gender difference in interpersonal coordination under the experimental conditions of the soccer experiment.

“Winning is not everything, it’s the only thing” (Overman, 1999). This famous quote, attributed to the American football player and coach Henry Russel Sanders, must be acknowledged, although the aesthetic appearance of a well-coordinated team play is pleasing to the eye. Therefore, we probe in the next step whether the scoring differences shown in Figure 1B are significant.

For the male teams, we detect a highly significant scoring advantage of at least one or two goals in the Sy-condition in comparison to the wR-mode and even more pronounced for the comparison of the Sy- and nS-conditions. For *D*≥3, the probability that goal differences between the Sy- and nS-condition will occur by chance is still less than 5%. From 8 events, 7 have been denoted in favor of the Sy-condition. Naturally, elevated scoring differences *D* occur less frequently, which influences the estimation of *p*-values for small samples. Hence, significance by trend decreases with increase in *D*. However, considering that for soccer games the scoring statistics is highly noise contaminated (Heuer et al., 2010; Tolan et al., 2013), which makes the outcome of a soccer game hard to predict (Skinner and Freeman, 2009), and that the participants have not practiced with headphones beforehand such that our results reflect the spontaneous reaction of the cRAS, we believe that the high significance of the goal statistics obtained for the male teams are astounding.

Like in the case of the connectivity index, for the female teams, no significant difference between the synchronous and non-synchronous conditions could be detected for any *D* shown in Figure 3. Counter-intuitively, for the comparisons with the wR-condition, *p*-values decrease with *D*. For*D*≥3, both *p*-values are below 0.05. In view of the results presented in Figure 2, the increased scoring rates of the female teams under acoustic stimulation might be provoked by decreased efficient team play and decreased coupling between players probably due to the obstructive influence of the headphones. The last statement is backed up by the observation that the number of goals per game decreases on average with the quality of the football league. (refer to, e.g., www.fussball.de).

We also tested for the influence of fatigue along the three thirds but did not measure any significant effect. To this end, we compared the connectivity values as well as the scoring rates and probe statistical equivalence by Mann-Whitney-Wilcoxon-rank test. No significant difference between the first and the last thirds could be detected. Furthermore, we repeated the statistical evaluation by employing parametric tests (namely the Student *t*-test and the paired *t*-test. For further information refer to Supplementary material) and obtained quantitatively similar results.

We conclude that the results presented so far are not due to a particular philosophy of the data analysis (parametric vs. nonparametric statistics) but reflect a genuine effect of (a) an improved interpersonal coordination under an adequate acoustic environment providing a common time frame, (b) a pronounced correlation between the connectivity index and the scoring rate for the male teams and (c) according to our knowledge a, so far, unobserved gender difference.

The question remains to be “which mechanism is responsible for the highly significant effect for the male teams and for which reason the female teams react so differently?”. It is well-known that there is an elevated functional connectivity between the auditory cortex and the motor cortex in human brains (Thaut et al., 1999; Chen et al., 2008; Altenmüller and Schlaug, 2013), which leads to entrainment effects of motor skills such that, e.g., locomotion gets spontaneously modified (Molinari et al., 2003). This finding seems to be independent of physical body features (MacDougall and Moore, 2005).

It was shown that a more similar movement dynamics facilitates prediction of future actions (Vesper et al., 2011), and it has been pointed out that joint action is promoted if subjects share similar features of their motor dynamics (Stowinski et al., 2016; TsanevaAtanasova, 2016). Hence, a possible explanation of the above scenario is that the motor entrainment in the Sy-condition causes a more similar movement dynamics of the 5 players, while in the nS-condition where each player perceives its personal acoustic pacemaker, one may suppose that motor entrainment provokes larger spreading of, e.g., stride frequencies. What if the tempo adjustment of the Sy-mode is not appropriate for female players? Then, it cannot be expected that the rhythm displayed at 140 bpm will promote team play, and that the stimulus might have a disturbing influence on interpersonal coordination. Of note, this argument holds solely if motor entrainment, provoked by the acoustic rhythm displayed at 140 bpm, promotes assimilation of the moving frequency of male but not female subjects. We tested for this effect in a first follow-up experiment.

Notwithstanding, it is doubtable if this principle applies to association football in a strict sense where players must adjust their motor performance according to the game situation they encounter and, may be the most important, according to the movement of the players of the opposing team and only partly to that of their own teammates. Their behavior is context-dependent (Turvey, 2007; Araújo and Davids, 2016). One may also observe that a defending player synchronizes his motion to a certain extent with that of the ball-leading one, especially in areas close to their own goal, to block the opponent’s way. Accordingly, this follow-up experiment clearly demonstrates that no assimilation of stride frequencies occurs because of motor entrainment (refer to Supplementary material). Hence, motor entrainment does not provide a possible mechanism capable of explaining the results observed in the soccer experiment. We provide empirical evidence for this conclusion in Supplementary material.

## The Stroop experiment

The acoustic rhythm neither presents some feedback nor is directly related to the performance of soccer players. Instead, it provides a common time frame for organizing efficient interpersonal coordination. It influences the level of attention and promotes anticipation skills and adaptation of own actions (Riess Jones et al., 2006; Keller et al., 2014). It was shown that the sensory motor system is involved in selective attention and anticipatory mechanism (Schubotz, 2007; Morillon et al., 2014), which in turn fosters interpersonal coordination because it supports the fine-tuning of self-actions with those of interaction partners (Riess Jones et al., 2006; Keller et al., 2014). Such rhythmic modulation of attention level (Riess Jones et al., 1981; Large and Riess Jones, 1999; Trapp et al., 2018) draws a dynamical picture of attention driven by, e.g., external acoustic stimuli (Escoffier et al., 2010; Bolger et al., 2013). Thus, a preferred tempo of acoustic rhythms improving (motor) cognition (Keller et al., 2014) would provide a strong indication for a resonance-like behavior of brain dynamics (Large, 2008) and affords a plausible explanation of the outcomes of the soccer experiment.

If the attention dynamics of the players could be synchronized, e.g., *via* synchronously displayed acoustic stimuli, this would positively influence the joint action of the players (Sebanz et al., 2006). The prerequisite for this is that acoustic rhythms can modulate the level of attention. To obtain empirical evidence that supports these theoretical considerations, we designed and conducted a follow-up experiment.

### Description of the Stroop experiment

To probe whether the level of attention can be influenced by acoustic rhythms and to explore whether we detect some tempo dependency, we used a modified version of the Stroop interference task known as the color-word matching Stroop task (Zysset et al., 2001). In this version of the Stroop test, the cognitive part is slightly more complicated, and the response is just by pressing a button. The participants had this test in the acoustic environment of the same rhythm used for the soccer experiment, but now we probe three different tempos, namely, 100, 140, and 180 bpm, while measuring the reaction timeΔ*t* of the participants.

In total, 60 male and 77 female subjects with mean age of  23.7 ± 3.5 and 23.5 ± 4.1, respectively, participated and performed a variant of the Stroop test already used elsewhere (Masataka and Perlovsky, 2013) while hearing the acoustic rhythms used in the soccer experiment with headphones. For each participant, we applied the rhythm with solely one tempo to avoid the influence of a probable memory effect.

The task consisted of 3 blocks. All of them were composed of two runs with 48 trials each. Both runs comprised 24 congruent and 24 incongruent randomized trials. The first run was named “neutral test” and worked as a control to estimate the reaction time of the participants for perception, reading, and pressing the response button. The second run, named “Stroop test,” was presented to estimate the Stroop effect, which is meant to represent the effect elicited by the incongruence of the features. The first and third blocks were presented without music, and the second block was performed while listening to the rhythm used in the soccer experiment displayed at one of the following tempi: 100, 140, or 180 bpm. Hence, the dependent variable was the reaction time, and the independent variable was the musical tempo. For the data analysis presented here, only incongruent trials were considered. For each tempo, a group of men and women was recruited. After the first analysis, we sorted the female participants according to the phase of their menstrual cycle, which was obtained by forward-counting, and we repeated the analysis separately for each group. The number of participants for each phase of the menstrual cycle is shown in Table 1.

**TABLE 1:** Number of male and female subjects participating in the Stroop-experiment under the influence of different tempi.

Tempo/bpm	Male	Female			
		Menstrual	Folicular	Ovulatory	Lutea
100	20	10	2	4	8
140	20	10	2	6	7
180	20	10	5	3	8

The female participants are listed separately for different stages of the menstrual cycle.

The quantitative comparison of the reaction times obtained for the different settings is performed by Mann-Whitney-Wilcoxon-rank test. Besides the estimated *p*-values, the median and borders of the 95% confidence interval of the samples are also reported to provide information about the effect of size. More details about the Stroop experiment can be found in the last part of Supplementary material.

## Results of the Stroop experiment

**FIGURE 4:**
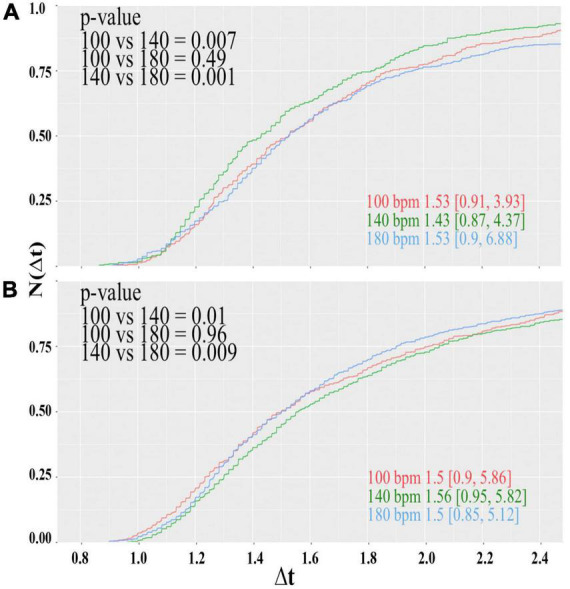
Cumulative distributions of the reaction times of the color-word matching Stroop task while the participants were under the influence of the acoustic rhythm used in the soccer experiment displayed at 100 (red), 140b (green), and 180 bpm (blue). Panels **(A,B)** show the results for the male and female subjects, respectively. Inserted numbers indicate the median value and borders of the 95% confidence interval. Furthermore, *p*-values for the pairwise comparison of the different samples according to the Mann-Whitney-Wilcoxon-rank test are indicated.

The results obtained for the reaction times of the participants are summarized in Figure 4.

For the male subjects (Figure 4A), we observe a clear preference for 140 bpm. The difference of the central values of the distribution obtained for 140 bpm and the others is of the order Δ*t*≈ 0.1 s, which connotes a decrease in the reaction time of about 6.5% and is highly significant. For the female subjects, on the other hand, the performance at 140 bpm is worse, but no clear difference between 100 and 180 bpm can be observed. By trend, reaction times obtained for 180 are slightly shorter given that the distribution for 100 bpm has a much longer tail toward largeΔ*t*; however, statistically, the central values of the distributions (Figure 4B) are indistinguishable.

A preferred tempo of the acoustic stimulus for male brains resembles a resonance-like behavior. It seems that neuronal circuits, which are responsible for attentional tasks, get entrained by the activity of neuronal ensembles that are processing acoustic information, a clear indication of the dynamical nature of attentional processes (Large and Riess Jones, 1999; Large, 2008). This behavior is like that of coupled, nonlinear oscillators where one dynamical unit triggers the response of the others (Vicente et al., 2008). If such resonance-like behavior is a generic principle for male brains, why do we not observe similar effects for female subjects albeit their preferred tempo might differ from 140 bpm. This last conjecture is based on indications that functional networks of acoustic information processing are different for men and women (Corsi-Cabrera et al., 2007).

**FIGURE 5:**
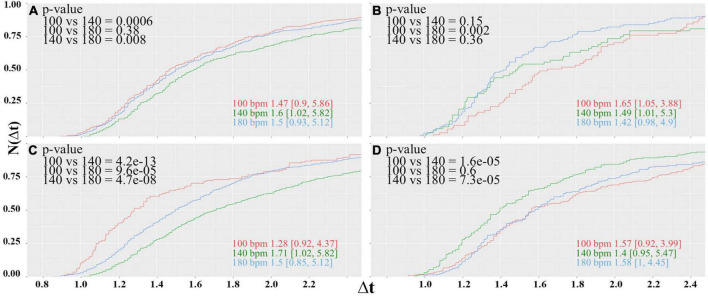
Same as Figure, but now the female subjects are divided according to the actual phase of their menstrual cycle. Cumulative distributions of reaction times of the female participants in the **(A)** menstrual, **(B)** folicular **(C)** ovulatory and **(D)** luteal phase

A possible explanation for this incongruity might be that neural activity is altered during the menstrual cycle (Solís Ortiz et al., 1994). The influence of sexual steroids in different areas of the cortex where estrogenic receptors moderate acetylcholine production, which in turn is involved in selective attention processes (Corsi-Cabrera et al., 2007), might provoke that hormonal changes during the menstrual cycle influence the outcome of the Stroop experiment. Furthermore, it was documented that women, within the first days of their menstrual cycle, show a significantly improved performance in the Stroop task than those within days 21 and 22 (Hatta and Nagaya, 2009), which correspond to the luteal phase. To probe such hormonal dependency, we divided the group of female participants according to their actual phase of the menstrual cycle (refer to Table 1). The results obtained for women in the menstrual, follicular, ovulation, and luteal phases are shown in Figure 5.

Under the new selection criteria, the picture changes drastically. The women in the menstrual phase represent the largest group of the female participants with 10 subjects for each tempo; hence, the results shown in Figure 4B are somewhat biased by this group. Like in the former case where all female participants form one single group, the performance at 140 bpm is worse, but now the cumulative distribution for 100 and 180 bpm has the same shape. The best performance is now observed when the rhythm is displayed at 100 bpm, although the difference between 100 and 180 bpm is only in trend. The median values differ by only 0.03 s and turns out to be insignificant.

The women in the folicular phase, on the other hand, show a slight preference for 180 bpm and worse performance when the rhythm is displayed at 100 bpm. The subjects in the ovulation phase again seem to prefer 100 bpm, and slowest reaction times are observed at 140 bpm. The effect size for this group is considerably large. Differences between central values of adjacent distributions are slightly larger than 0.2 s and connote an effect of about 14%. However, the results of both groups should be taken with caution, because the number of participants for each tempo of 3 to 6 subjects is considerably low (Table 1).

Finally, the group in the luteal phase had 8 to 9 participants for each tempo. For this group, a clear preference for 140 bpm can be seen, while distribution functions obtained for 100 and 180 bpm are statistically equivalent. The central value of the cumulative probability distribution for 140 bpm is about 0.16 s lower than the values observed for the lower and faster tempos, yielding an effect of 11%, which proves to be highly significant.

We acknowledge that the group size when different phases of the menstrual cycle are distinguished is by far not sufficient for drawing definite conclusions. Therefore, we propose to repeat the whole Stroop experiment in a future study in a more controlled manner. Only women between 20 and 30 years old that do not take any contraceptive drugs, do not conduct any kind of hormone therapy, and are regular in the sense that their menstrual cycle does not vary more than plus/minus 1 day should be considered to improve the control of hormonal balance. With this inclusion criteria, a sufficiently high number of participants for each tempo should be recruited. Furthermore, to obtain a more detailed picture of a possible resonance phenomenon, more tempi should also be probed.

However, even the results presented in Figures 4, 5 provide at least a strong indication for a kind of entrainment of attention processes with a preferred tempo of the acoustic rhythm. In addition, this tempo preference seems to depend on the hormonal balance of the subjects. Given that this parameter was not controlled in the soccer experiment, one cannot expect to find a tempo preference for the female teams.

## Discussion

The empirical data gained from the soccer experiment show a pronounced response of the male teams to a certain rhythmic acoustic stimulus. A successful play in terms of scoring rate could be significantly enhanced, a result that correlates strongly with the connectivity measure introduced by us. At the same time, it seems at a first glance that females do not show any sensitivity to their acoustic environment in terms of the connectivity of their team play or their efficiency regarding scoring rates. At this point, two questions arise: the first is “why does a certain rhythm, or rather a rhythm displayed to all teammates at a certain tempo, improve or worsen the team play of the male teams although one might expect that there is no interference between listening to music and performance on the soccer field? The improvement observed for the synchronous setting is surprising given the obstructive influence of earphones while doing team sport, because it hinders the acoustical coupling between teammates. In this context, it was shown that the intention of the game opponent can be inferred from the sound of his motor actions. This information can be used as a guide for one’s own reactions (Camponogara et al., 2017). Such action-related auditory cues (Cañal-Bruland et al., 2018) are widely suppressed when RAS is supplied. The second question is “why do the female teams behave strikingly different?”. Seemingly, the results obtained for synchronous and non-synchronous do not differ statistically for women.

The results of the Stroop experiment, however, imply that the same resonance-like mechanism is active in the male and female brains. An acoustic rhythm displayed at an appropriate (preferred) tempo enhances the level of attention of subjects, which in turn positively influences interpersonal coordination. This result is gender-independent, which indicates that the principal mechanism of men and women is the same as could be expected beforehand. However, in addition, we encounter a striking dependence on hormonal balance, an uncontrollable parameter in the soccer experiment. Hence, the Stroop experiment provides a consistent explanation for the initially surprising result. The increased connectivity of the male teams in comparison to the non-synchronous setting and, as a consequence, the improved scoring rate can be attributed to the improved attentional level provoked by the synchronously displayed rhythm at 140 bpm, the preferred tempo for the male subjects in the Stroop experiment. Furthermore, it was impossible to control for the seemingly strong dependence of the preferred tempo on the hormonal level in the soccer experiment. Based on these results, it cannot be expected that the female teams will show any effect.

However, is it tenable to think of a resonance phenomenon of the brain dynamics? Its hierarchical structure supports the hypothesis of a resonating brain. Sets of few neurons form microcircuits, which are assembled to larger modules up to the constitution of macroscopic structures like the thalamo-cortical system. All these hierarchically intertwined modules are feedback loops, which are oscillating with their preferred rhythm (Buzsaki, 2006). A hierarchical organization has also been observed for oscillations of the functional brain network where delta rhythm modulates activity of faster frequency bands up to the gamma band by phase-amplitude locking (Lakatos et al., 2008, 2013). In this context, it is important to note that cortical activity is entrained to acoustical rhythms (Fujioka et al., 2009; Large et al., 2015). Delta activity may synchronize to acoustic stimuli (Will and Berg, 2007; Stefanics et al., 2010; Gomez-Ramirez et al., 2011; Nozaradan, 2014), which in turn trigger theta rhythms (Will and Berg, 2007) as well as activity in the alpha, beta- and lower gamma-bands (Will and Berg, 2007; Gomez-Ramirez et al., 2011). Cyclic modulation of alpha activity is associated with effective social coordination (Naem et al., 2012) and attention processes in a way that alpha activity acts as an inhibitor of distracters while gamma activity focuses on attention (Ward, 2003).

Hence, it seems that the brain operates generally in a rhythmic mode, with frequency preferences in terms of nonlinear resonances of dynamical units acting on different scales of space and time where low frequency activity may control the dynamics measured in higher frequency bands, which in turn are responsible for a variety of cognitive processes and attention. Delta activity may act as an instrument for attentional selection (Schroeder and Lakatos, 2008). In this picture, entrainment of neuronal oscillating activity (Lakatos et al., 2008; Nozaradan, 2014) may also directly induce an improvement in cognitive skills (Xing et al., 2016) without involving motor areas. Thus, the brain can be understood as a finely orchestrated assembly of dynamical units able to resonate.

Musical stimuli have an immediate influence on attention and cognitive abilities (Lakatos et al., 2008, 2013), which may result in a kind of attentional entrainment that decays only gradually (Trapp et al., 2018). Importantly, the reaction time in a highly demanding visual attention task varies notably when subjects hear the same acoustic stimulus at different tempi (Amezcua et al., 2005; Husain et al., 2002) and may modulate the perception of physical exertion (Martins-Almeida et al., 2015; Patania et al., 2020). Therefore, it is also conceivable that appropriate adjustment of the tempo of an acoustic rhythm is imperative in the present context, as could be substantiated by the Stroop experiment.

In this spirit and given that the maximal time lag by which the synchronously displayed rhythm is perceived by the players is below 10 ms, it seems that the common acoustic framework not only elevates but also synchronizes the level of attention of the subjects and equivalently promotes interpersonal coordination of the whole team in a self-organized manner. In this dynamical picture, synchronized attention dynamics provides a common ground for joint action (Sebanz et al., 2006) and promotes synergy in a team (Riley et al., 2011; Araújo and Davids, 2016). This explains the significant positive effect measured for the male teams and simultaneously the initially counter-intuitive results for the female teams, which are possibly provoked by our finding that the preferred tempo of the female subjects apparently changes during the menstrual cycle.

At this point, we like to underscore that the results obtained for women in different phases of their menstrual cycle are still not conclusive but should be understood as a first experimental indication for this finding. To provide a substantial empirical finding, the number of well-selected participants in each group and for each tempo should be increased notably, which goes beyond the scope of this study and will be treated in a forthcoming study. However, this viewpoint is supported by the fact that functional brain networks during music perception are strikingly disparate between male and female subjects (Corsi-Cabrera et al., 2007; Flores-Gutiérrez et al., 2009). Therefore, it is plausible to assume that preferred frequencies of active functional networks are notably different between genders because of dependency on hormonal balance (Solís Ortiz et al., 1994; Corsi-Cabrera et al., 2007; Hatta and Nagaya, 2009). However, we should also take into consideration that the 140 bpm of the synchronous setting was optimized for male 400-m racers; thus, in principle, it could be inadequate for women.

The results obtained so far clearly indicate that (a) the connectivity and, hence, efficiency (goal statistics) of a soccer team is improved in the Sy setting compared to the nS condition, and that (b) the attention level of the male teams improves when exposed to the rhythm displayed at 140 bpm. However, it might be interesting to investigate in future experiments whether the positive influence of cRAS can compensate for the reduced acoustic coupling between players (Camponogara et al., 2017; Cañal-Bruland et al., 2018). Thus, although the results of the male subjects in the Stroop experiment clearly indicate a resonance phenomenon, it would be interesting in future experiments to have a team play in the Sy condition against a team without acoustic stimulation (wR).

Besides the theoretical considerations of underlying brain dynamics, this study might have additionally a major practical impact on training strategies. The long-term effect of music therapy, even for a short-lasting therapy duration (Thaut et al., 1999; Altenmüller and Schlaug, 2013), provides an optimistic projection in this direction, leading toward the conclusion that interpersonal coordination may also improve in a competing situation of a league game if a team is accustomed to practice systematically in an appropriate acoustic environment. The last statement holds to be true not only for aesthetic aspects of team sports but also quantitatively in terms of scoring rate.

## Conclusion

We discover a basic mechanism of interpersonal coordination and succeeded to integrate the observed phenomena into an established theoretical framework, namely, the dynamic attention theory in combination with a resonance-like tempo preference of rhythmic acoustic stimuli. Furthermore, we unravel a, so far, unknown gender difference; additionally, our findings may open new avenues for development of novel training strategies in team sports.

It is all about rhythm!

## Data availability statement

The datasets presented in this study can be found in online repositories. The data of the soccer experiment are available at: https://github.com/antonietamg/Soccer_experiment_data, the Stroop experiment can be found in: https://github.com/antonietamg/Stroop_Data_2022, and the stride frequency experiment are located at: https://github.com/antonietamg/Rhythm_and_Sync_Data.

## Ethics statement

The studies involving human participants were reviewed and approved by Ethic Committee of the Autonomous University of Morelos (UAEM), Nu. of Folio 240918-05. All participants were informed about details concerning the execution and evaluation of our experiments and all participants were aware that they could stop participating at any time without any consequences. They gave their written consent to their participation and in case of minors; we got the permission of the legal guardians. None of the experiments conducted by us put any participant in danger or got harmed. For instance, the soccer players followed a well-known training scheme they are used to doing in their practice sessions while the Stroop experiment is a common attention test in psychological studies.

## Author contributions

MAM formulated the initial idea of this research and the first experiment. MAM, AF, MH, AE, and GS designed the first experiment. AE and GS acquired the technical equipment. MAM and AF conducted the first experiment and revised the video tapes. MH produced the acoustic stimuli. IG-M and MFM designed the stride frequency experiment. IG-M conducted the stride frequency experiment. MAM and MFM conducted the corresponding statistical analysis and wrote the manuscript. MFM and AM-G designed the Stroop experiment and made the Figures. AM-G conducted the Stroop experiment and did the respective statistical analysis in accordance with MFM. MC-C contributed essentially to the interpretation of the results. MAM, MC-C, AM-G, and MFM constructed the psychological model. All authors revised the manuscript and took part in the discussion of the results.
