# Supplementary material for: How to orchestrate a soccer team: Generalized synchronization promoted by rhythmic acoustic stimuli
Source: Front Hum Neurosci. 2022 Jul 29;16:909939. doi: 10.3389/fnhum.2022.909939 (PMC9372544; doi:10.3389/fnhum.2022.909939)
Supplement: Supplementary file 1 [file Data_Sheet_1.docx]

**Supplementary Material of the Article:**

**How to Orchestrate a Soccer Team**

**-Generalized Synchronization Promoted by Rhythmic Acoustic Stimuli-**

Manfred A. Müller^1^, Antonieta Martínez-Guerrero^2^, María Corsi-Cabrera^3,4^, Alfred O. Effenberg^5^, Armin Friedrich^1^, Ignacio Garcia-Madrid^6^, Matthias Hornschuh^7^, Gerd Schmitz^5^, Markus F. Müller^1,8,9*^

^1^Centro Internacional de Ciencias, A.C., Avenida Universidad S/N, 62131 Cuernavaca, Morelos, México

^2^ Instituto de Ciencias Básicas y Aplicadas, Universidad Autónoma del Estado de Morelos, 62209 Cuernavaca, Morelos, México

^3^Sleep Laboratory, Faculty of Psychology, Universidad Nacional Autónoma de México, México

^4^Institute of Neurobiology, Universidad Nacional Autónoma de México, Campus Juriquilla, Queretaro**,** México

^5^Leibniz Universität Hannover, Institut für Sportwissenschaft, Am Moritzwinkel 6, 30559, Hannover, Germany

^6^Posgrado en Ciencias Sociales, Facultad de Estudios Superiores de Cuautla, Universidad Autónoma del Estado de Morelos. Carretera México-Oaxaca 218, Plan de Ayala, 62743 Cuautla, Morelos, México.

^7^Stiftung Universität Hildesheim, Institut für Musik und Musikwissenschaft, Kulturcampus Domäne Marienburg, Universitätsplatz 1, 31141 Hildesheim, Germany

^8^Centro de Investigación en Ciencias, Universidad Autónoma del Estado de Morelos, 62209 Cuernavaca, Morelos, México

^9^Centro de Ciencias de la Complejidad, Universidad Nacional Autónoma de México, México, D.F., México

*Correspondence to: [muellerm@uaem.mx](mailto:muellerm@uaem.mx)

|  | Passes | | Goals | | N_0_ | |
| --- | --- | --- | --- | --- | --- | --- |
|  | Men | Women | Men | Women | Men | Women |
| wR | 11.7 | 13 | 0.65 | 0.78 | 0.36 | 0.59 |
| Sy | 12.2 | 13.2 | 0.84 | 0.96 | 0.32 | 0.62 |
| nS | 11 | 12.8 | 0.6 | 0.94 | 0.48 | 0.47 |

**Table S1:** Comparison between male and female teams using statistics normalised to playing time.

**Quantitative Evaluation of the Empirical Data of the Soccer Experiment using Parametric Statistics:**

To substantiate our analysis presented in the main text we also applied a parametric test framework. In the case of the connectivity values estimated from the number of passes and ball contacts we employed the two-sample student t-test.

**FIGURE S1**

**Figure S1**: Probabilities that normalized connectivity samples obtained for the synchronous and the non-synchronous condition have statistically equivalent mean values. P-values are shown in a logarithmic scale. Blue bars refer to results obtained for male teams, pink coloured bars to those obtained for female teams. Horizontal black lines indicate the 1 and 5% significance level. **A**: for increasing “n” successively short pass-sequences are excluded; **B**: for decreasing “n” successively long pass-sequences are excluded.

Note that we retain almost quantitatively the same p-values as those obtained by using the Mann-Whitney-Wilcoxon-rank test. Only for very large or very short pass sequences probability estimations are notable above the 5% significance level. As argued in the main text such tendency should be expected.

For the scoring statistics we employ a paired t-test, where pairs of scoring rates are formed for the same team, playing under different conditions. The corresponding results are displayed in Figure S2. Again, we reproduce almost quantitatively the results obtained for the non-parametric statistics, namely in the present case the estimates for the binomial-distribution.

**FIGURE S2**

**Figure S2:** Probabilities that scoring rates occur by chance according to a two-sample paired t-test. Panel **A** displays results obtained for male teams, panel **B** those for female teams for minimal goal differences between conditions of at least$D\geq1,2$or 3. Compared are wR with Sy-conditions (light blue/pink), wR with nS-condition (blue/pink) and Sy with nS-condition (dark blue/pink).

We also provide audio files of the acoustic stimuli.

Audio file 1 to 6 correspond to 119, 133, 140, 147, 154 and 161 bpm.

**The Stride Frequency Experiment:**

We probe if motor-entrainment may provide a consistent explanation for the results obtained in the soccer experiment. To this end we designed a follow-up experiment where subjects’ stride frequency was measured while walking with and without hearing the acoustical rhythms already used in the synchronous setting of the soccer experiment and with and without using a distracter (leading a ball while walking) to reveal whether the rhythmic acoustic environment leads to an assimilation of stride frequencies.

18 male and 18 female (mean age is $24.9\pm3.4$ and $24.3\pm5.4$ years respectively) are advised to walk one by one 28m (the length of a basketball court) on their preferred stride frequency, without and with RAS of 140bpm. In each condition each subject walked three times. The same experiment was repeated but now people had to lead a ball, which serves as a kind of distracter. This is to create a situation more akin to the first experiment, where the subjects primarily concentrate on playing soccer while perceiving the acoustic environment in a more unconscious way. Here stride and kick frequencies must be adjusted, such that a pure motor entrainment, as reported in multiple studies (Thaut et al., 1999, Molinari et al., 2003), gets hindered.

However, in case that the mechanism advised in (Stowinski et al., 2016, Keller et al., 2014) has been effective in the soccer experiment one would expect that spontaneous stride frequencies of subjects get more similar due to motor-entrainment (Chen et al., 2008) when walking under the influence of the RAS displayed at 140bpm.

Hence, the purpose of this follow-up experiment is to compare motor-entrainment effects with and without a distracter (leading a ball while walking) and to reveal whether the rhythmic acoustic environment leads to an assimilation of stride frequencies.

In Figure S3 the cumulative distributions of spontaneous stride frequencies for male and female subjects with and without leading a ball, with and without the influence of an acoustic environment of the same rhythm used in experiment one displayed with 140bpm.

**FIGURE S3**

**Figure S3:** Cumulative probability distribution of the stride frequency of male (panel **A** and **C**) and female subjects (panel **B** and **D**), which are advised to walk on their preferred stride frequency, with (dark blue/pink) and without RAS displayed with 140bpm (light blue/pink), with (panels **C** and **D**) and without (panel **A** and **B**) leading a ball. Each subject walked in each condition three times. Inserted numbers quantify the medians and the 95% confidence intervals of the samples.

Spontaneous stride frequencies of females are on the average systematically higher than those of male subjects. Furthermore, curves obtained with RAS are shifted to the right due to motor-entrainment, although people do not spontaneously synchronize to the external stimulus. However, distributions do not get narrower when people walk with the 140bpm rhythm. On the contrary, in this case the 95% confidence intervals get slightly larger. Hence, the mechanism that an assimilation of movement rhythms improves interpersonal coordination (Stowinski et al., 2016, Keller et al., 2014) is not active in the present case.

**The Stroop Experiment:**

The Stroop test applied in the second follow-up experiment contains three modes: neutral congruent and non-congruent. A visual representation of each mode is provided by Figure S4.

**FIGURE S4**

**Figure S4:** Representation of the neutral, congruent, and non-congruent mode of the Stroop experiment. On the right-hand side, the time lapses between the representation of each stimulus are given.

Note, also the neutral mode may contain visual dissonant stimuli if the colour of the three “X” does not coincide with the colour name below. We adopted this scheme from earlier works (Zysset et al., 2001, Masataka and Perlovsky, 2013). For each mode the reaction time has been measured.

In Figure S5 we present the comparison of the cumulative distribution of the reaction time measured for the neural mode and the incongruent mode of the Stroop test.

One observes a striking difference between both modes. The difference of the median values reaches values above 0.3 seconds, an enhancement of the performance of about 25 to 30%. Corresponding p-values of a pairwise comparison of the neutral and the incongruent condition separately for the different tempi are below${10}^{-15}$. For the comparison of the different tempi of the neutral mode we encounter shortest reaction times when the stimulus is displayed with 140bpm for male subjects as well as a significantly improved performance for women within the luteal phase. This finding is in accordance with the results encountered for the incongruent condition, where for both groups a clear preference for the 140bpm tempo has been measured. However, in overall statistics when all female subjects are considered irrespective in which phase of their menstrual cycle they are, performance is worst for 140pm. This is true for the neutral as well as the incongruent condition.

**FIGURE S5**

**Figure S5:** Comparison of the neutral and incongruent condition. Cumulative distributions of the reaction times of male and female subjects performing the Stroop test under the influence of the different tempi are shown for the neutral (dashed lines) and the incongruent condition (solid lines). Panel **A** displays cumulative distribution of reaction times for men, panel **B** for all women, panel **C** and **D** differentiate between female subjects within the first three days of the menstrual cycle or within the luteal phase respectively. In coloured letters median values and the borders of the 95% confidence interval are given. Furthermore, the p-values of a pairwise comparison of the different modes according to the Mann-Whitney-Wilcoxon-rank test are included.

Furthermore, also women within the first three days of the menstrual cycle performed significantly worst in the neutral mode, when the rhythm was displayed with 140bpm and a tendency in favour of 100bpm can be observed for the incongruent condition for this group. Note, the neutral condition serves in the present context as a control, viz. the fact that for male subjects and for women within the first three days of their menstrual cycle we observe a significant improvement for the incongruent mode under the influence of 140bpm and 100bpm rhythm respectively, but no significant improvement for the same tempi in the neutral mode, implies directly that attention level is positively affected by the acoustic background. However, given that for women within the luteal phase both, neutral and incongruent condition show shortest reaction times when the rhythm is displayed with 140bpm suggests that also other processes (like e.g., reading ability) than attention are influenced.

**References:**

Chen J.L., Penhune V.B., Zatorre R.J., 2008, *Listening to Musical Rhythms Recruits Motor Regions of the Brain*, Cerebral Cortex **18**, 2844-2854

Keller PE, Novembre G, Hove MJ 2014 *Rhythm in Joint Action: Psychological and Neurophysiological Mechanisms for Real-Time Interpersonal Coordination*, Phil. Trans. R. Soc. B **369**, 20130394

Masataka N, Perlovsky L 2013 *Cognitive interference can be mitigated by consonant music and facilitated by dissonant music*, Scientific Reports **3**, 1-6

Molinari M, Leggioi MG, de Martin M, Cerasa A, Thaut MH 2003 *Neurobiology of Rhythmic Motor Entrainment*, Ann. N.Y. Acad., Sci. 999, 313-321

Thaut MH, Kenyon GP, Schauer ML, McIntosh GC 1999 *The Connection between Rhythmicity and Brain Function,Iimplications for Therapy of Movement Disorders, IEEE Engineering in Medicine and Biology*, 101-108

Stowinski P, Zhai C, Alderisio F, Salesse R, Gueugnon M, Marin L, Bardy BZ, di Bernardo M,

Zysset S, Müller K, Lohmann G, Cramon DV 2001 *Color-Word Matching Stroop Task: Separating Interference and Response Conflict*. NeuroImage **13**, 29-36
