# Supplementary figures and images for: How to orchestrate a soccer team: Generalized synchronization promoted by rhythmic acoustic stimuli
Source: Front Hum Neurosci. 2022 Jul 29;16:909939. doi: 10.3389/fnhum.2022.909939 (PMC9372544; doi:10.3389/fnhum.2022.909939)

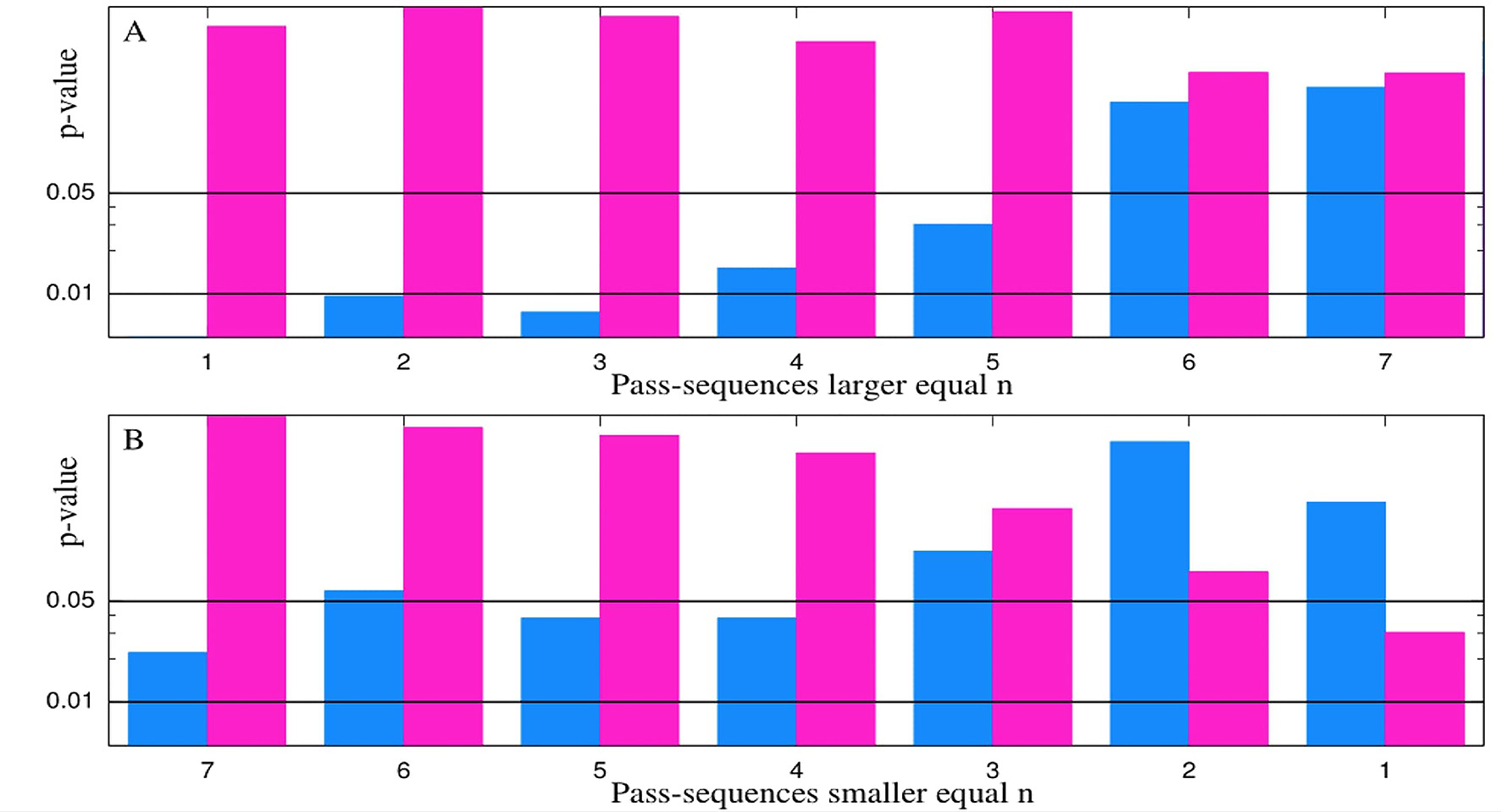

Supplement: Supplementary file 8 [file Image_1.JPEG]

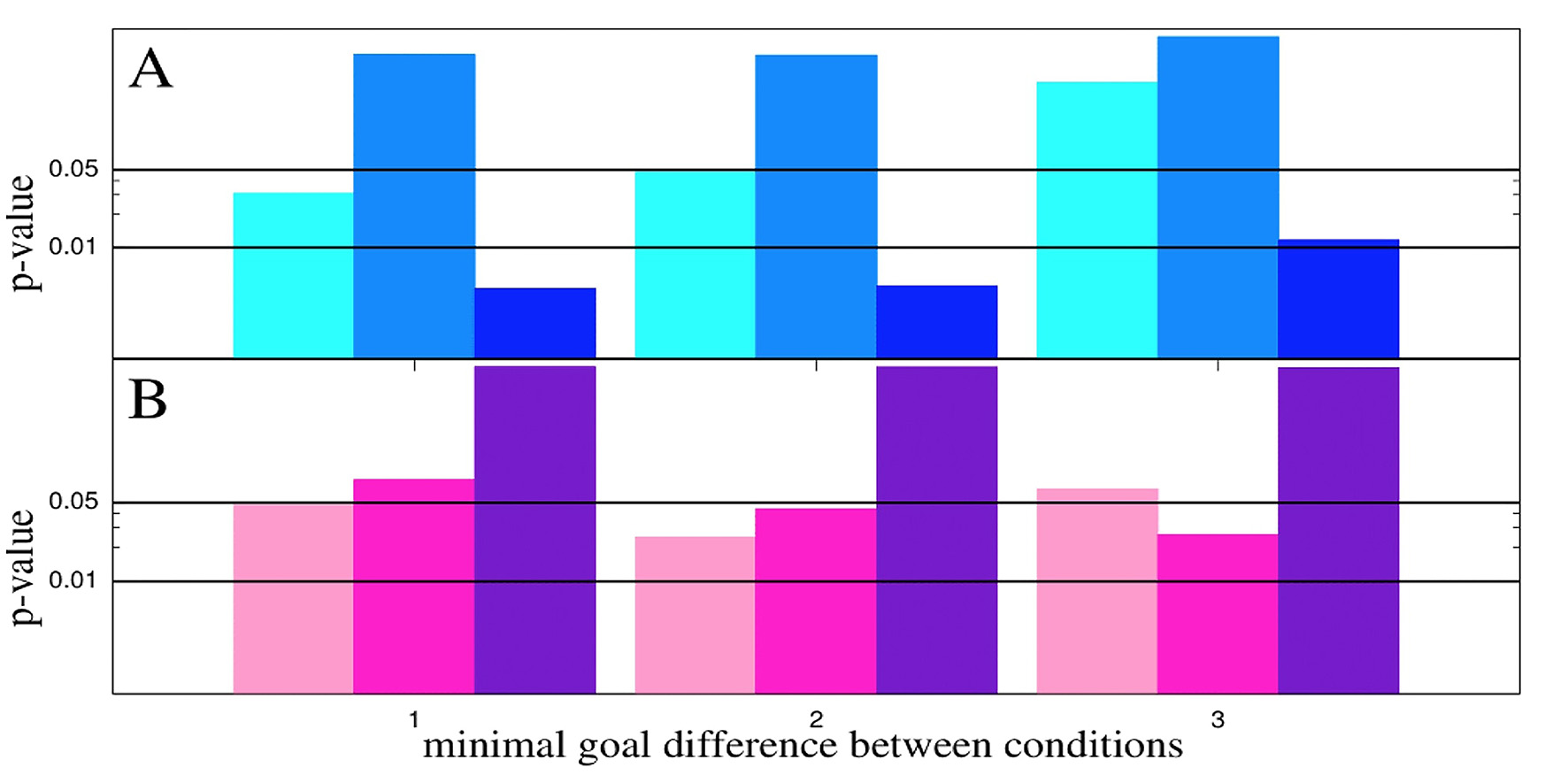

Supplement: Supplementary file 9 [file Image_2.JPEG]

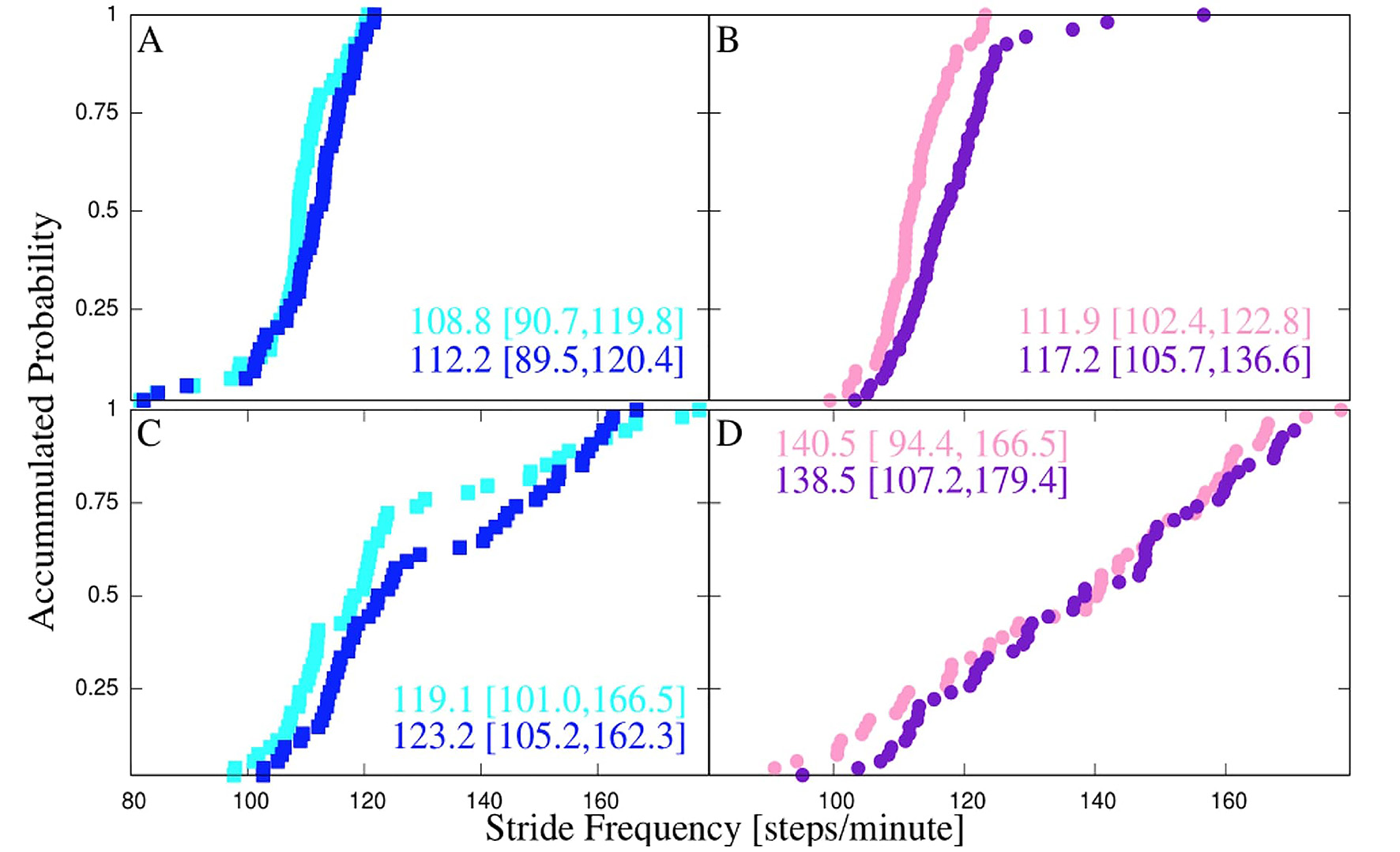

Supplement: Supplementary file 10 [file Image_3.JPEG]

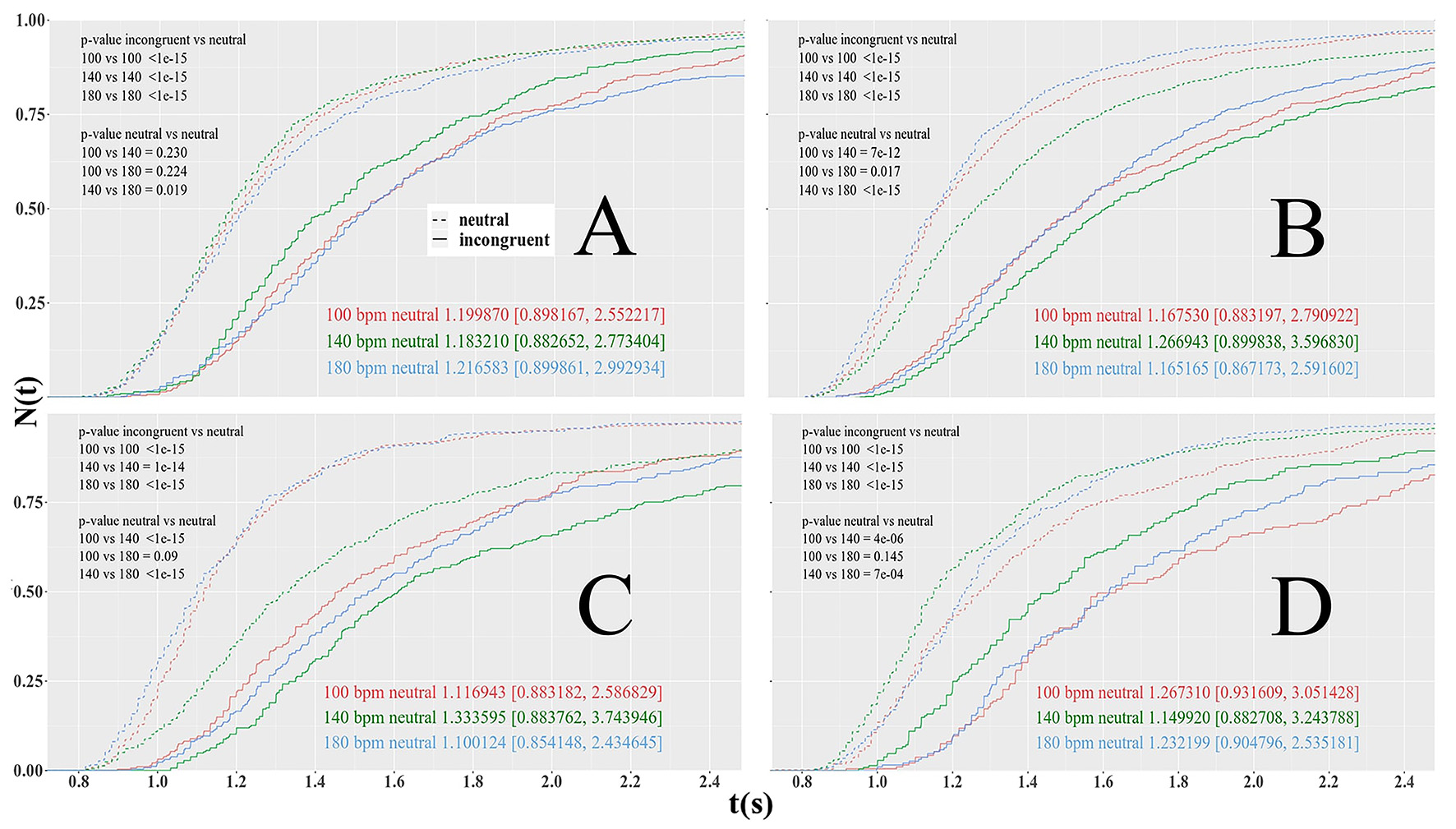

Supplement: Supplementary file 11 [file Image_4.JPEG]
